# Characteristics and trends of unintentional injuries among children and adolescents in Kunshan, China: a hospital-based retrospective study, 2018–2023

**DOI:** 10.3389/fpubh.2025.1606347

**Published:** 2025-06-11

**Authors:** Li Shen, Yafang Hua, Chunfang Gu, Rong Tao, Yuwei Zhang, Bin Huang, Mingqing Yuan, Weibing Wang, Jian Huang, Zhiping Li

**Affiliations:** ^1^Department of Clinical Pharmacy, National Children’s Medical Center, Children’s Hospital of Fudan University, Shanghai, China; ^2^Department of Pharmacy, Suzhou Hospital, Affiliated Hospital of Medical School, Nanjing University, Suzhou, China; ^3^Department of Nursing, Kunshan Woman and Children’s Healthcare Hospital, Children’s Hospital of Fudan University Kunshan Branch, Kunshan, China; ^4^Department of Public Health, Kunshan Woman and Children’s Healthcare Hospital, Children’s Hospital of Fudan University Kunshan Branch, Kunshan, China; ^5^Department of Medical Administration, Kunshan Woman and Children’s Healthcare Hospital, Children’s Hospital of Fudan University Kunshan Branch, Kunshan, China; ^6^Department of Pharmacy, Kunshan Woman and Children’s Healthcare Hospital, Children’s Hospital of Fudan University Kunshan Branch, Kunshan, China; ^7^Key Laboratory of Public Health Safety, Ministry of Education, Department of Epidemiology, School of Public Health, Fudan University, Shanghai, China; ^8^Department of Hospital Administration, Kunshan Woman and Children’s Healthcare Hospital, Children’s Hospital of Fudan University Kunshan Branch, Kunshan, China

**Keywords:** unintentional injury, children and adolescents, epidemiological trends, time series analysis, ARIMA model

## Abstract

**Objective:**

This study aimed to characterize the epidemiological trends and mechanisms of pediatric unintentional injuries in Kunshan, China (2018–2023) and to develop time series models to predict future trends.

**Methods:**

A retrospective analysis was conducted on 77,379 pediatric unintentional injury cases, stratified by age, gender, and injury categories. Subgroup analyses targeted children under 5 years. To adjust for pandemic-related disruptions, separate comparisons between 2018 and 2023 were performed. Time series analysis employed an ARIMA model, with model selection based on information criteria and residual diagnostics, and a non-COVID-19 dataset (2018, 2019, and 2023) for forecasting future trends.

**Results:**

Males constituted 62.76% of cases, with a mean age of 5.37 ± 3.55 years. The primary age groups were 3–6 years and 6–12 years, which accounted for 60% of the total population. Falls (21.36%) and transport injuries (4.00%) predominated, with limbs being the most injured body part (59.08%). Contusions/abrasions (41.54%) and sprains/strains (31.21%) were common. Subgroup analysis was performed in children under 5 years old, with 22,110 being males (57.5%) and 16,291 being females (42.5%). Among this group, falls and burns were identified as the most frequent incidents. Unintentional injury cases decreased significantly during COVID-19 (2020–2022). The refined ARIMA(1,1,2) (0,1,1)[12] model, excluding pandemic effects, achieved a mean absolute percentage error of 6.46% while revealing seasonal patterns and predicting a slight downward trend for 2024–2026.

**Conclusion:**

Pediatric unintentional injuries in Kunshan exhibited gender and age-specific patterns, with COVID-19 altering injury profiles. The ARIMA model can capture the seasonal patterns of unintentional injuries to a certain extent, facilitating public health planning and intervention strategies.

## 1 Introduction

Unintentional injuries were the primary cause of death among children and play a major role in childhood morbidity, as well as contributing significantly to long-term disability and healthcare expenses (1). Approximately 1 million children succumb to unintentional injuries every year worldwide (2). The economic burden of pediatric unintentional injuries was equally worrisome, with estimates showing a significant impact in terms of healthcare costs, potential future income, and the overall quality of life for the young (3). The impact of childhood injuries on families was complex, particularly in relation to mental health. Research showed that family members, especially parents, would suffer from significant psychological distress and strain, such as feelings of anxiety and depression (4). These mental health challenges often arise from the intense anxiety and ongoing concern parents face following their child’s injury. Common causes of unintentional injuries include falls, road traffic incidents, drowning, poisoning, and burns. Although extensive research has documented these causes and their impacts, critical problems persist in the uneven distribution of injury risks and preventive measures across different socioeconomic groups and geographic regions (5–7). In China, rapid industrialization and urban expansion have led to unique challenges in maintaining child safety. Unintentional injury remained the top cause of death for children under five, with approximately 26,600 fatalities, accounting for almost 15% of all child deaths (8). The likelihood of children experiencing unintentional injuries was positively correlated with economic development, family income, and education level (9). Unintentional pediatric injuries exhibit varying causes and characteristics across different regions. Child deaths in the United States were associated with unintentional firearm injuries, significantly increasing the likelihood of hospitalization (10). Nevertheless, by implementing effective prevention measures, the majority of unintentional injuries could be avoided and managed (11).

Exploring the causes and characteristics of unintentional injury among children was essential for identifying prevention strategies. By studying real-world data, we can analyze the time series trend of childhood injuries and gain valuable insights to help reduce the prevalence of these injuries. By delving into the specifics of these injuries, this study aimed to shed light on underlying causes and potential preventative measures. The autoregressive integrated moving average (ARIMA) model, consisting of autoregression (AR), integration (I), and moving average (MA), was widely recognized as a classic approach for time series analysis (12). ARIMA models were designed to describe time-dependent data points by capturing patterns such as trends and seasonality; for example, they could assess the impact of meteorological and environmental factors on pediatric wheezing (13). ARIMA models have been applied in forecasting dengue fever incidences and influenza morbidity, illustrating their utility in public health scenarios where accurate predictions are essential for management strategies (14, 15). Additionally, the ARIMA framework can be extended to include seasonal variations, termed Seasonal ARIMA (SARIMA), enhancing its applicability in situations where seasonality is significant (16).

This study aimed to characterize the trends of pediatric unintentional injuries in Kunshan from 2018 to 2023, with a specific focus on age and gender stratification. Through an in-depth analysis of over 77,000 pediatric unintentional injury cases, our study identified the most prevalent injury types and characteristic patterns across different age groups. Through the construction of an ARIMA time series model, we comprehensively analyzed the developmental trends of pediatric unintentional injuries in Kunshan, including a forecast of injury trends for the subsequent 3 years. These findings may offer critical evidence-based insights for developing targeted pediatric unintentional injury prevention strategies and informing public health policy.

## 2 Materials and methods

### 2.1 Study design

Patients under 18 years of age who presented to the outpatient or emergency departments of Kunshan Woman and Children’s Healthcare Hospital between January 2018 and December 2023 and were diagnosed with unintentional injuries were included in this retrospective cohort study. Unintentional injuries, defined as physical harm resulting from accidental events, such as falls, burns, poisonings, drownings, or road traffic incidents-were distinguished from injuries resulting from intentional violence or self-harm. The study protocol was approved by the Ethics Committee of Kunshan Woman and Children’s Healthcare Hospital (No. 2024-03-001-H001) in accordance with the Declaration of Helsinki. Waiver of informed consent was granted based on the retrospective design, complete anonymization of medical records, and absence of additional risk exposure to participants (17).

The International Classification of Diseases, 10th Revision (ICD-10) was a coding system utilized by healthcare professionals globally to categorize and code all diagnoses, symptoms, and procedures. In this study, unintentional injuries, as classified by ICD-10 codes, included falls (W00–W19), transport accidents (V01–V99), accidental drowning and submersion (W65–W74), accidental poisoning (T36–T65), exposure to smoke, fire, and flames (X00–X09), accidental suffocation and strangulation (W75–W84), animal bites (Y00–Y34), as well as other miscellaneous injuries (X10–X43, X50–X59, W20–W64, W85–W94). The data collection process involved following the inclusion and exclusion criteria, demographics, characteristics, and outcomes.

The study focused on the cases that were initially diagnosed as unintentional injuries in medical facilities such as emergency rooms, trauma centers, and outpatient clinics. The collection did not encompass cases where the same injury in a medical machine was revisited, given that the electronic medical records system was employed to retrieve the medical records.

### 2.2 Data analysis

A descriptive analysis was conducted to present an overview of the characteristics of the study subjects. Continuous variables were summarized using the mean and standard deviation (SD), providing a measure of central tendency and dispersion, respectively. For categorical variables, percentages were used to describe the distribution of these variables. To explore the general characteristics of pediatric unintentional injuries, the data were stratified by gender, age groups, different quarters, and years, which allowed for a comparison of means and percentages across different subgroups. Furthermore, the analysis extended to categorize injuries based on their nature, and the study can assess the frequency of various types of injuries (e.g., falls, burns) in children of different ages and quarters.

In-depth, the ARIMA model was employed to assess the temporal trends of unintentional injuries. The Akaike Information Criterion (AIC) and Bayesian Information Criterion (BIC) were frequently utilized for comparing different ARIMA models (18). Generally, the model with the lowest AIC and BIC values was considered to have the best performance (19).

The analyses were performed via EmpowerRCH (X&Y Solutions Inc., Boston, MA, United States), with R 4.2.3 or GraphpadPrism used for figure visualization. A *p*-value less than 0.05 was utilized to determine the level of significance.

## 3 Results

### 3.1 Basic characteristics

**Figure 1:**
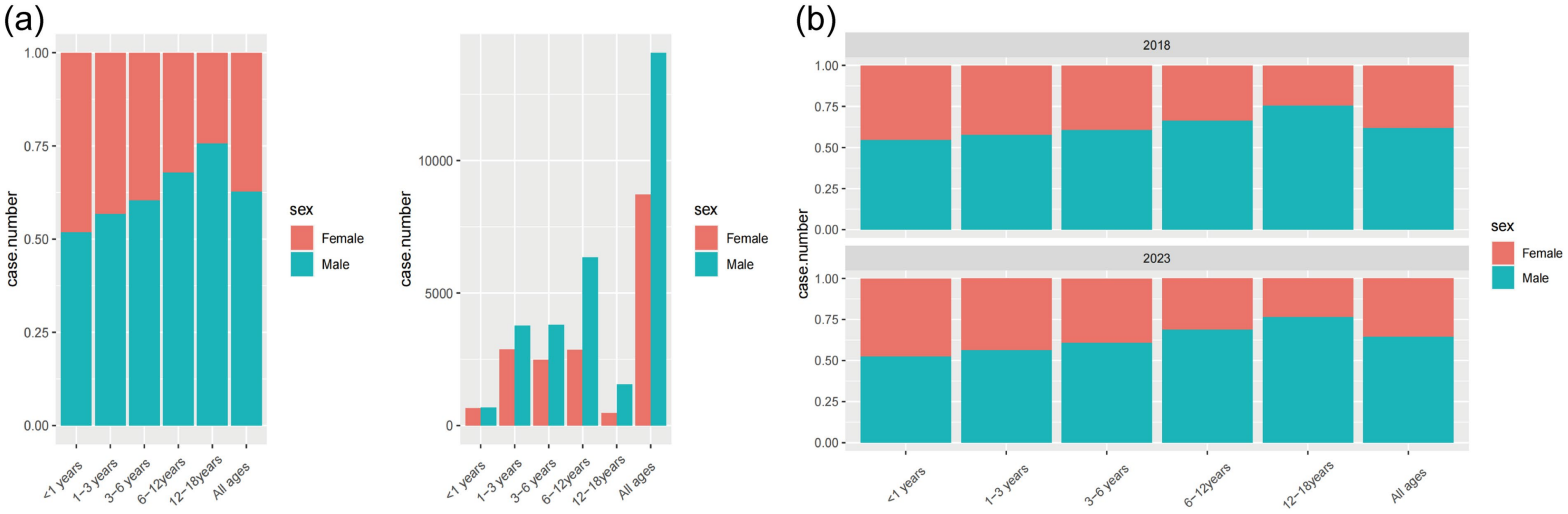
Frequency of unintentional injuries in six age groups. **(a)** The distribution of unintentional injuries in different age groups and genders. **(b)** The distribution of unintentional injuries across age groups and genders in 2018 and 2023.

Throughout the study period, a total of 77,379 individuals experienced unintentional injuries, with 48,562 (62.76%) being males and 28,817 (37.24%) females. The mean age was 5.37 ± 3.55 years, with males being older (5.73 ± 3.63 years) compared to females (4.76 ± 3.34 years). The basic characteristics can be seen in Table 1 and Figure 1. Age distribution showed that the majority of individuals were in the age groups 3–6 years (26.85%) and 6–12 years (35.49%). In terms of unintentional injury categories, the most common was “fall” (21.36%), followed by “transport” injuries (4.00%). The limbs were the most frequently injured body part (59.08%). The predominant nature of injuries included contusions/abrasions (41.54%), sprains/strains (31.21%), and fractures (13.62%). The majority of individuals received treatment in outpatient/emergency departments (96.59%), with a small proportion hospitalized (2.88%). Surgical intervention was required in 1.27% of cases. Data distribution across the years 2018 to 2023 showed a relatively even spread, with a decrease in 2020 and 2021, likely due to the COVID-19 pandemic. Monthly and quarterly distributions were consistent, without significant fluctuations.

**Table 1:** Demographic and clinical characteristics of pediatric unintentional injuries.

Terms	Male, *n*(%) *N* = 48,562	Female, *n*(%) *N* = 28,817	Total *N* = 77,379
Age, years	5.73 ± 3.63	4.76 ± 3.34	5.37 ± 3.55
Age (groups), years			
<1	2,190 (2.83)	2,032 (2.63)	4,222 (5.46)
≥1 to <3	11,019 (14.24)	8,396 (10.85)	19,415 (25.09)
≥3 to <6	12,548 (16.22)	8,226 (10.63)	20,774 (26.85)
≥6 to <12	18,637 (24.09)	8,823 (11.40)	27,460 (35.49)
≥12 to <18	4,168 (5.39)	1,340 (1.73)	5,508 (7.12)
Injury category			
Transport	1,848 (2.39)	1,244 (1.61)	3,092 (4.00)
Fall	10,417 (13.46)	6,109 (7.89)	16,526 (21.36)
Blunt/heavy object	724 (0.94)	407 (0.53)	1,131 (1.46)
Firearm	1 (0.00)	0 (0.00)	1 (0.00)
Knife/sharp object	1,272 (1.64)	633 (0.82)	1,905 (2.46)
Fire, heat, and hot substances	1,402 (1.81)	1,020 (1.32)	2,422 (3.13)
Asphyxiation	3 (0.00)	1 (0.00)	4 (0.00)
Drowning	34 (0.04)	19 (0.02)	53 (0.07)
Poisoning	516 (0.67)	524 (0.68)	1,040 (1.34)
Animal contact	487 (0.63)	352 (0.45)	839 (1.08)
Unknown/others	31,858 (41.17)	18,508 (23.92)	50,366 (65.09)
Position of injury			
Head	17,718 (22.90)	9,743 (12.59)	27,461 (35.49)
Body	2,018 (2.61)	1,116 (1.44)	3,134 (4.05)
Limbs	28,282 (36.55)	17,432 (22.53)	45,714 (59.08)
Not available	544 (0.70)	526 (0.68)	1,070 (1.38)
Nature of injury			
Fracture	6,954 (8.99)	3,588 (4.64)	10,542 (13.62)
Sprain/strain	13,944 (18.02)	10,206 (13.19)	24,150 (31.21)
Sharp object injury/bite/open wound	4,563 (5.90)	2,201 (2.84)	6,764 (8.74)
Contusion	20,949 (27.07)	11,193 (14.47)	32,142 (41.54)
Burn/scald	1,374 (1.78)	989 (1.28)	2,363 (3.05)
Concussion/brain contusion	55 (0.07)	38 (0.05)	93 (0.12)
Organ system injury	164 (0.21)	71 (0.09)	235 (0.30)
Adverse effects of medical treatment	496 (0.64)	496 (0.64)	992 (1.28)
Others	63 (0.08)	35 (0.05)	98 (0.13)
Department			
Outpatient/emergency	46,834 (60.53)	27,907 (36.07)	74,741 (96.59)
Rescue	217 (0.28)	193 (0.25)	410 (0.53)
Hospitalized	1,511 (1.95)	717 (0.93)	2,228 (2.88)
Year			
2018	12,064 (15.59)	7,461 (9.64)	19,525 (25.23)
2019	14,073 (18.19)	8,719 (11.27)	22,792 (29.46)
2020	1,261 (1.63)	751 (0.97)	2,012 (2.60)
2021	972 (1.26)	645 (0.83)	1,617 (2.09)
2022	6,164 (7.97)	3,512 (4.54)	9,676 (12.50)
2023	14,028 (18.13)	7,729 (9.99)	21,757 (28.12)
Quarter			
Q1	7,949 (10.27)	4,916 (6.35)	12,865 (16.63)
Q2	13,651 (17.64)	7,813 (10.10)	21,464 (27.74)
Q3	13,681 (17.68)	8,530 (11.02)	22,211 (28.70)
Q4	13,281 (17.16)	7,558 (9.77)	20,839 (26.93)
Surgery			
No	47,890 (61.89)	28,503 (36.84)	76,393 (98.73)
Yes	672 (0.87)	314 (0.41)	986 (1.27)
Death			
No	48,555 (62.75)	28,809 (37.23)	77,364 (99.98)
Yes	7 (0.009)	8 (0.01)	15 (0.02)

### 3.2 Mechanisms of unintentional injuries

**Figure 2:**
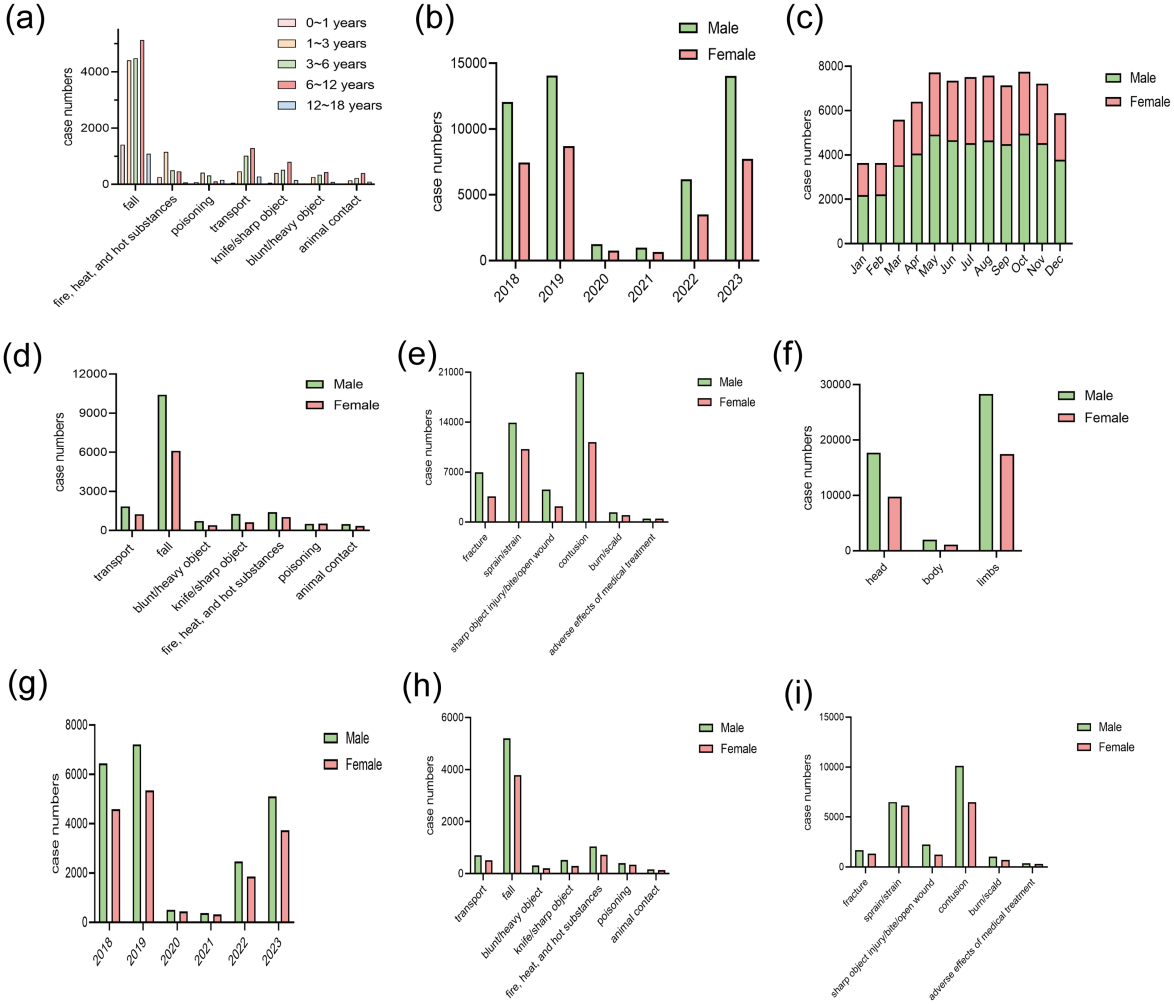
Characteristics of unintentional injuries. **(a)** The top 7 frequencies of injuries in different age groups. **(b)** The distribution of unintentional injuries in different years. **(c)** The distribution of unintentional injuries in different months. **(d)** The top 7 frequencies of injuries in different genders. **(e)** The top 6 frequencies of injury nature. **(f)** The injury position. **(g)** The distribution of unintentional injuries in children under five in different years. **(h)** The top 7 frequency of injuries in children under five. **(i)** The top 6 frequencies of injury nature in children under five.

Subgroup analysis was performed in children under 5 years old, with the characteristics detailed in Table 2 and depicted in Figures 2a–i. There was a total of 38,401 children under 5 years old, comprising 22,110 males (57.5%) and 16,291 females (42.5%). The mean age was significantly different between genders, with males averaging 2.42 ± 1.07 years and females 2.30 ± 1.08 years (*p* < 0.001). The distribution of cases over the years 2018 to 2023 showed a significant difference (*p* = 0.008), with the highest proportions in 2019 (18.80% males, 13.94% females) and 2023 (13.31% males, 9.73% females). The nature of injuries varied significantly between genders (*p* < 0.001), with contusions/abrasions being the most common in both males (26.39%) and females (16.86%), followed by sprains/strains (16.93% males, 16.02% females). Injury categories showed that fall was the most common for both genders (13.53% males, and 9.88% females). In addition, to comprehensively describe the characteristics of unintentional injuries across different age groups (under 1 year, 1–3 years, 3–6 years, 6–12 years, and 12 years and older), Supplementary Table S1 provided a detailed stratified analysis.

**Table 2:** Characteristics of unintentional injuries in children under 5 years old.

Gender	Male, *n* (%) *N* = 22,110	Female, *n* (%) *N* = 16,291	*P*-value
Age, years	2.42 ± 1.07	2.30 ± 1.08	<0.001
Year			0.008
2018	6,434 (16.75)	4,580 (11.93)	
2019	7,218 (18.80)	5,352 (13.94)	
2020	504 (1.31)	445 (1.16)	
2021	372 (0.97)	320 (0.83)	
2022	2,471 (6.43)	1,858 (4.84)	
2023	5,111 (13.31)	3,736 (9.73)	
Nature of injury			<0.001
Fracture	1,692 (4.41)	1,347 (3.51)	
Sprain/strain	6,501 (16.93)	6,152 (16.02)	
Sharp object injury/bite/open wound	2,271 (5.91)	1,227 (3.20)	
Contusion	10,134 (26.39)	6,475 (16.86)	
Burn/scald	1,025 (2.67)	708 (1.84)	
Concussion/brain contusion	21 (0.05)	14 (0.04)	
Organ system injury	44 (0.11)	27 (0.07)	
Adverse effects of medical treatment	387 (1.01)	320 (0.83)	
Others	35 (0.09)	21 (0.05)	
Position of injury			<0.001
Head	10,189 (26.53)	6,655 (17.33)	
Body	820 (2.14)	592 (1.54)	
Limbs	10,683 (27.82)	8,710 (22.68)	
Not available	418 (1.09)	334 (0.87)	
Injury category			<0.001
Transport	709 (1.85)	510 (1.33)	
Fall	5,196 (13.53)	3,794 (9.88)	
Blunt/heavy object	313 (0.82)	205 (0.53)	
Knife/sharp object	523 (1.36)	291 (0.76)	
Fire, heat, and hot substances	1,043 (2.72)	724 (1.89)	
Asphyxiation	2 (0.01)	1 (0.00)	
Drowning	20 (0.05)	9 (0.02)	
Poisoning	402 (1.05)	343 (0.89)	
Animal contact	162 (0.42)	138 (0.36)	
Unknown/others	13,740 (35.78)	10,276 (26.76)	
Department			0.297
Outpatient/emergency	21,728 (56.58)	15,991 (41.64)	
Rescue	138 (0.36)	94 (0.24)	
Hospitalized	244 (0.64)	206 (0.54)	
Surgery			0.082
No	22,026 (57.36)	16,210 (42.21)	
Yes	84 (0.22)	81 (0.21)	

To counteract the repercussions of the COVID-19 pandemic on unintentional injuries, we performed separate analyses for cases in 2018 and 2023, as outlined in Table 3. The table analyzes the trends of unintentional injuries among 41,282 individuals, comparing data from 2018 (*n* = 19,525) and 2023 (*n* = 21,757). Gender distribution showed that the proportion of males increased from 61.79% in 2018 to 64.48% in 2023, and females decreased from 38.21 to 35.52%. Injury categories experienced significant changes (*p* < 0.001). The proportion of injuries caused by falls decreased from 21.81% in 2018 to 15.54% in 2023, while animal contact injuries rose from 65.72 to 72.81%. Notably, drowning injuries increased dramatically from 0.08% in 2018 to 1.87% in 2023. Overall, there was a gradual increase trend in unintentional injuries from 2018 to 2023.

**Table 3:** Trends of unintentional injuries in 2018 and 2023.

Year	2018, *n* (%) *N* = 19,525	2023, *n* (%) *N* = 21,757	*P*-value
Age, years	4.78 ± 3.34	6.07 ± 3.63	<0.001
Gender			<0.001
Male	12,064 (61.79)	14,028 (64.48)	
Female	7,461 (38.21)	7,729 (35.52)	
Injury category			<0.001
Transport	840 (4.30)	740 (3.40)	
Fall	4,259 (21.81)	3,380 (15.54)	
Blunt/heavy object	251 (1.29)	101 (0.46)	
Firearm	436 (2.23)	424 (1.95)	
Knife/sharp object	717 (3.67)	705 (3.24)	
Fire, heat, and hot substances	1 (0.01)	1 (0.00)	
Asphyxiation	17 (0.09)	4 (0.02)	
Drowning	16 (0.08)	406 (1.87)	
Poisoning	156 (0.80)	155 (0.71)	
Animal contact	12,832 (65.72)	15,841 (72.81)	
Position of injury			<0.001
Head	6,333 (32.44)	8,840 (40.63)	
Body	460 (2.36)	629 (2.89)	
Limbs	12,698 (65.03)	11,869 (54.55)	
Not available	34 (0.17)	419 (1.93)	

### 3.3 ARIMA model

**Figure 3:**
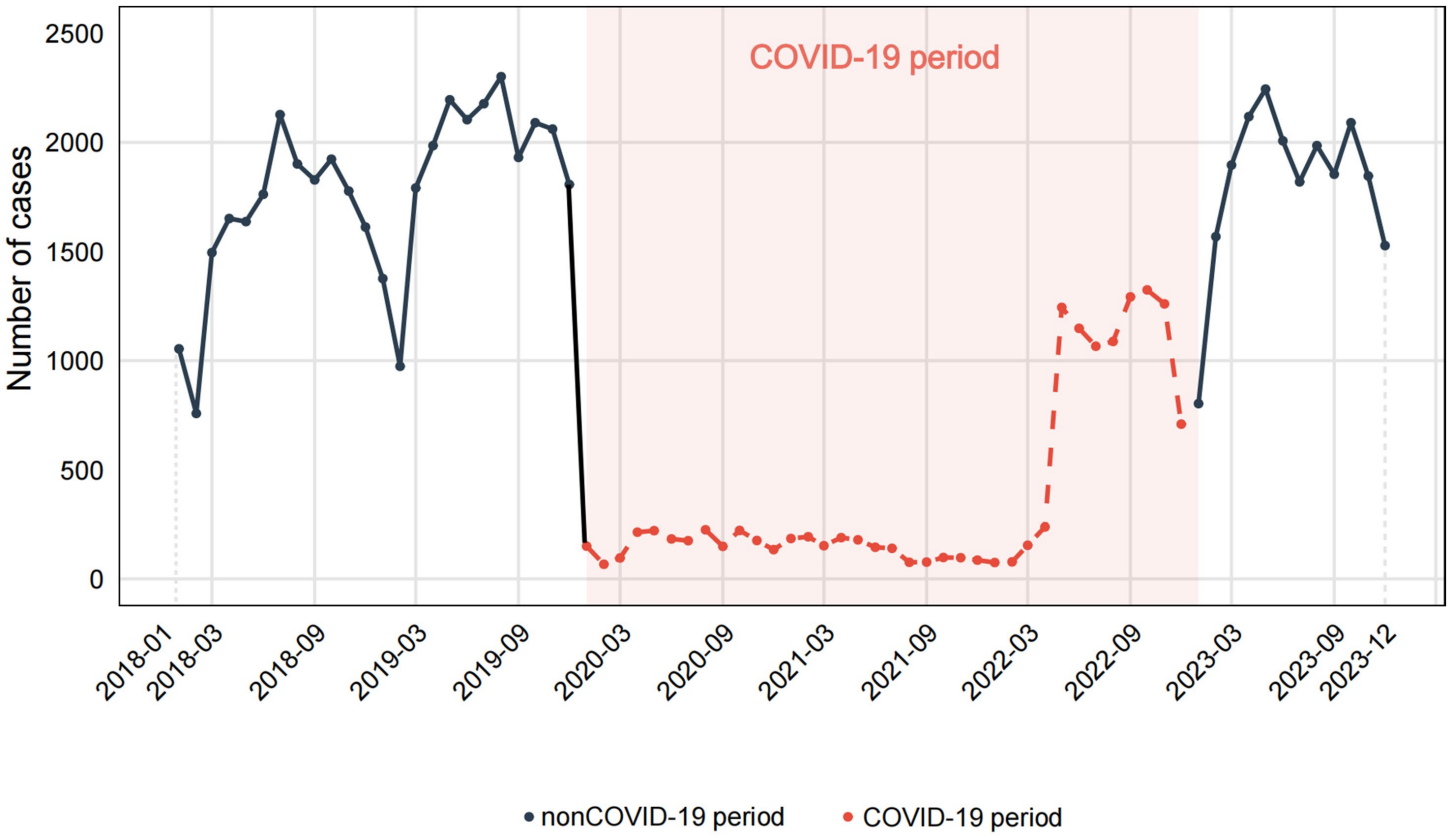
Temporal Trends of Childhood Unintentional Injuries in Kunshan, 2018–2023.

Time series analysis of 2018–2023 unintentional injury data showed initial nonstationarity (Augmented Dickey-Fuller test, *p* = 0.73) (Figure 3), which was improved through first-order differencing. Through information criterion minimization, ARIMA(0,1,0)(1,0,0)[12] was identified as the optimal model (AIC = 1022.23, BIC = 1026.75), with a mean absolute percentage error (MAPE) of 37.37% and a coefficient of variation of 29.33%. While residuals showed no significant autocorrelation (Ljung-Box *p* = 0.97), they demonstrated significant deviation from normality (Shapiro–Wilk *p* < 0.001), attributable to COVID-19 period disruptions.

**Figure 4:**
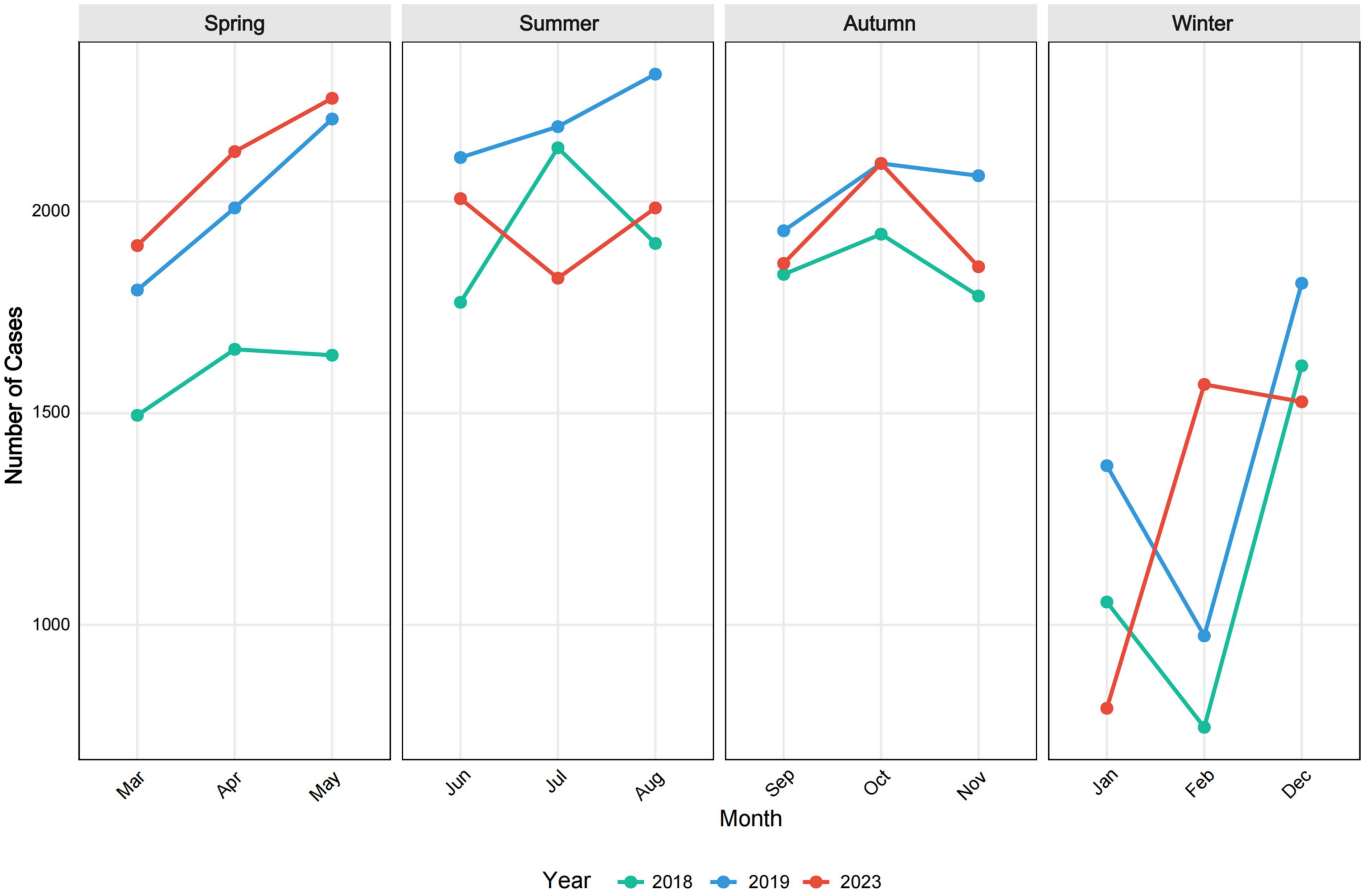
Seasonal Patterns in Childhood Unintentional Injuries in Kunshan.

**Figure 5:**
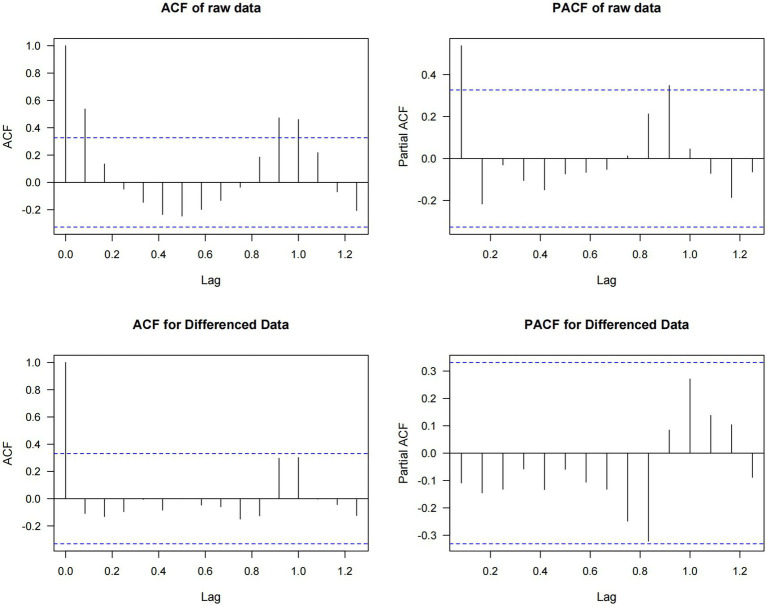
Autocorrelation and partial autocorrelation functions of original and first-order differenced time series.

**Figure 6:**
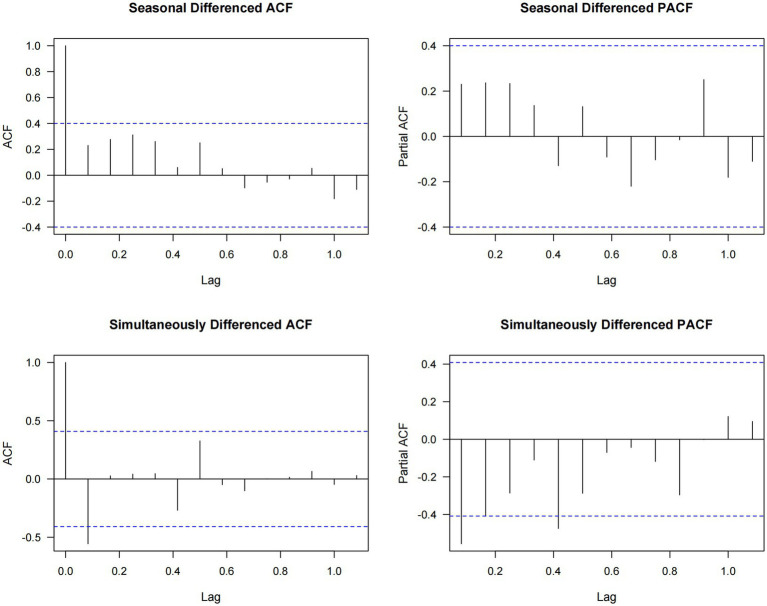
Autocorrelation and partial autocorrelation functions of seasonally and simultaneously differenced time series.

To account for the effects of COVID-19, we extracted data from 2018, 2019, and 2023 to construct a non-COVID-19 dataset for further analysis. Seasonal patterns of childhood unintentional injuries in Kunshan for the years 2018, 2019, and 2023 were illustrated in Figure 4. Across all 3 years, data showed a progressive rise in spring, a peak in summer or early autumn, and exhibited the most variability in winter. However, February consistently recorded the lowest case numbers throughout the year across all study periods. Figure 5 indicated that the raw data exhibits prominent positive autocorrelation at lag 1 and multiple significant lags, which is substantially weakened after applying differencing, leaving only partially significant correlations at certain lags. Figure 6 further presented the effects of seasonal and simultaneous differencing on the ACF and PACF, with simultaneous differencing producing a time series that was closest to stationarity. Based on the differencing analysis, the ARIMA modeling approach identified ARIMA(1,1,2)(0,1,1)[12] as the optimal model for pediatric unintentional injury data based on information criterion minimization, with an AIC of 328.34, and a BIC of 334.02. In the model, autoregressive (AR1 = 0.75), moving average (MA1 = −1.88, MA2 = 1.00), and seasonal moving average (SMA1 = 0.02) parameters were included. Excluding the pandemic-affected period significantly improved the model’s performance compared to using the complete dataset. It achieved a mean absolute percentage error of 6.46% and a mean absolute error of 93.68, indicating a more reliable characterization and stronger predictive capacity. Therefore, we employed this model to forecast pediatric unintentional injury cases in Kunshan for the upcoming three-year period.

**Figure 7:**
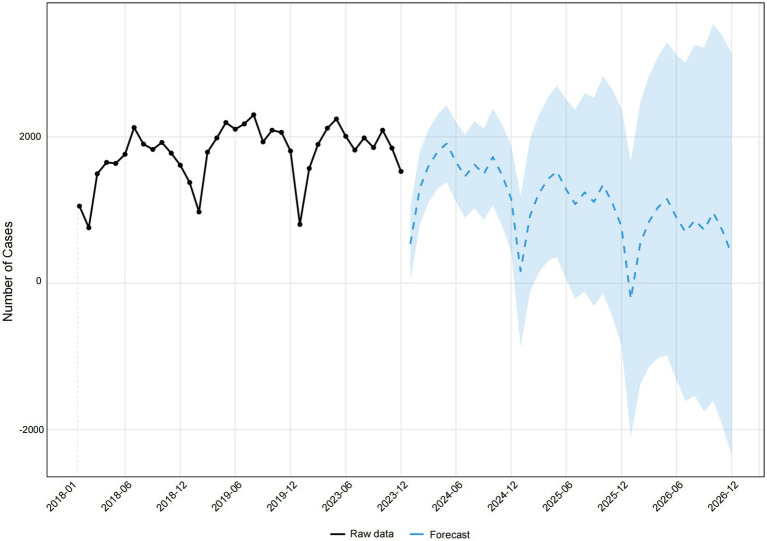
Forecasting Pediatric Unintentional Injuries in Kunshan, 2024–2026.

The time series forecasting of pediatric unintentional injuries in Kunshan for 2024–2026 was depicted in Figure 7, utilizing the ARIMA(1,1,2)(0,1,1)[12] model. The non-COVID-19 dataset demonstrated similar seasonal patterns with annual fluctuations, maintaining approximately 1,500–2,200 cases per month with periodic troughs appearing consistently in certain months. The forecast suggested continued seasonal fluctuations and a slight downward trend in the next three years, but increasing uncertainty was evident from the widening confidence intervals.

## 4 Discussion

Throughout this longitudinal analysis, we identified variations in the causes and characteristics of unintentional injuries among individuals aged 0–18 years, based on their age group and gender. The majority of unintentional injuries occurred in children aged 3–6 years and 6–12 years, with over 60% of cases reported in these age brackets. Our results indicated a persistent gender disparity in injury occurrence, with males consistently more prone to injuries than females. This observation aligns with previous studies suggesting that behavioral differences, possibly influenced by social and cultural norms, contribute to higher risk-taking activities among boys (20). The fact that falls were the primary cause of injuries, particularly among children under 5 years old, highlights the changing risk environment that came with children’s growing motor abilities and independence. Unintentional injuries related to poisons occurred almost equally among males and females. The common population presenting to the emergency department consisted of children under 12 years old who had been poisoned by medications (21). Nearly one-third of the children admitted for accidental poisoning were due to drug intoxication (22). Among the reasons for pediatric medication errors highlighted in a cross-sectional survey, the lack of medication knowledge among parents and caregivers was frequently mentioned (23).

The time series revealed a significant decrease in the number of unintentional injuries from 2020 to 2022; however, there was a notable increase in 2023. The trends were consistent with the impact of the COVID-19 pandemic. The first report of COVID-19 was in December 2019, and by 2022, solutions for COVID-19 in China had reached a normal stage (24). Due to stay-at-home policies and the shift to online learning (25), activity levels could decrease, resulting in fewer unintentional injuries. The utilization of ARIMA models further allowed us to describe the trends of unintentional injuries and assess the impact of interventions like the changes in public health policy during the COVID-19 pandemic.

Inevitably, the findings in this study had several limitations. During the study period, the COVID-19 pandemic had a substantial impact, leading to an anomalous decline in unintentional injury data between 2020 and 2022. Although a more accurate model was constructed by extracting non-pandemic period data from 2018, 2019, and 2023, this selective approach may limit the ability to fully capture long-term trends. Furthermore, our study employed a hospital-based retrospective analysis using medical records from the Kunshan Woman and Children’s Healthcare Hospital. One limitation of this study was that several injuries were categorized as “unknown/others,” reflecting the incompleteness of the data in the electronic medical record system. While providing a comprehensive analysis of unintentional injury characteristics and trends among children, the design unavoidably excluded cases not seeking medical care or treated at alternative facilities, potentially introducing selection bias and limiting the comprehensive understanding of regional injury patterns. Our future research will adopt a prospective multi-center approach to address these limitations. We aim to expand beyond mere injury types and numbers, implementing standardized assessment tools and establishing long-term tracking mechanisms to analyze key indicators such as hospitalization duration, medical costs, recovery status, and long-term effects. By collaborating with multiple medical institutions, we seek to construct a more comprehensive child injury database that enables more precise and nuanced insights for public health interventions.

As mentioned above, there has been a rise in the number of unintentional injuries among children. The results indicated that falls and traffic accidents constitute the predominant injury types among children in Kunshan - a city noted for its significant migrant population. This demographic characteristic necessitates targeted educational interventions for effective accident prevention. In response to the potential gaps in current child safety prevention strategies, we are developing a comprehensive, multi-dimensional intervention system. Specifically, we will establish a collaborative mechanism involving government, medical institutions, schools, and families, develop hierarchical safety education training programs, and leverage the resource advantages of the Yangtze River Delta region to develop standardized and replicable child safety intervention models. Within the volunteer network, we will set up tiered training and incentive mechanisms to ensure the breadth and depth of information dissemination. In platform construction, we plan to introduce big data and artificial intelligence technologies to achieve real-time analysis and precise warning of injury data. Simultaneously, we will establish a cross-departmental linkage response mechanism, enabling education, health, emergency, and other departments to intervene quickly and collaboratively against potential risks. These strategies aim not only to reduce the incidence of child accidental injuries but also to construct a comprehensive, systematic child safety protection network, providing replicable and scalable practical models for regional child safety governance.

## 5 Conclusion

In this study, we explored the causes and characteristics of unintentional injuries in children across various age groups and genders, serving as a foundation for future research and interventions aimed at mitigating these injuries. In order to reduce the risk of admission, it was important to implement preventive measures to decrease transport and fall injury, which had been shown to have a significantly higher risk. The government should pay great attention to the increase in unintentional injuries to children and adolescents in Kunshan from 2018 to 2023. Future research should focus on refining these predictive models with prospective study data integration. To reduce unintentional injuries in children, it is essential to strengthen successful strategies and customize policies and programs to address particular demographics.

## Data Availability

The original contributions presented in the study are included in the article/Supplementary material, further inquiries can be directed to the corresponding authors.
