# Supplementary material for: Characteristics and trends of unintentional injuries among children and adolescents in Kunshan, China: a hospital-based retrospective study, 2018–2023
Source: Front Public Health. 2025 Jun 11;13:1606347. doi: 10.3389/fpubh.2025.1606347 (PMC12187780; doi:10.3389/fpubh.2025.1606347)
Supplement: Supplementary file 1 [file Table_1.docx]

**Supplementary Table 1** Characteristics of unintentional injuries in children among different age groups

| **Characteristics** | <1, year | >=1, <3, year | >=3, <6, year | >=6, <12, year | >=12, year | P value |
| --- | --- | --- | --- | --- | --- | --- |
| **Gender** |  |  |  |  |  | <0.001 |
| Male | 2190 (51.87%) | 11019 (56.76%) | 12548 (60.40%) | 18637 (67.87%) | 4168 (75.67%) |  |
| Female | 2032 (48.13%) | 8396 (43.24%) | 8226 (39.60%) | 8823 (32.13%) | 1340 (24.33%) |  |
| **Nature of injury** |  |  |  |  |  | <0.001 |
| fracture | 167 (3.96%) | 1263 (6.51%) | 2590 (12.47%) | 4990 (18.17%) | 1532 (27.81%) |  |
| sprain/strain | 1293 (30.63%) | 6874 (35.41%) | 6148 (29.59%) | 8165 (29.73%) | 1670 (30.32%) |  |
| sharp object injury/bite/open wound | 143 (3.39%) | 1782 (9.18%) | 2174 (10.47%) | 2329 (8.48%) | 336 (6.10%) |  |
| contusion | 2283 (54.07%) | 7902 (40.70%) | 8983 (43.24%) | 11261 (41.01%) | 1713 (31.10%) |  |
| burn/scald | 249 (5.90%) | 1135 (5.85%) | 482 (2.32%) | 433 (1.58%) | 64 (1.16%) |  |
| concussion/brain contusion | 3 (0.07%) | 17 (0.09%) | 22 (0.11%) | 44 (0.16%) | 7 (0.13%) |  |
| organ system injury | 19 (0.45%) | 14 (0.07%) | 56 (0.27%) | 119 (0.43%) | 27 (0.49%) |  |
| adverse effects of medical treatment | 54 (1.28%) | 399 (2.06%) | 294 (1.42%) | 91 (0.33%) | 154 (2.80%) |  |
| others | 11 (0.26%) | 29 (0.15%) | 25 (0.12%) | 28 (0.10%) | 5 (0.09%) |  |
| **Position of injury** |  |  |  |  |  | <0.001 |
| head | 2392 (56.66%) | 8381 (43.17%) | 8235 (39.64%) | 7626 (27.77%) | 827 (15.01%) |  |
| body | 182 (4.31%) | 678 (3.49%) | 780 (3.75%) | 1233 (4.49%) | 261 (4.74%) |  |
| limbs | 1583 (37.49%) | 9933 (51.16%) | 11451 (55.12%) | 18490 (67.33%) | 4257 (77.29%) |  |
| not available | 65 (1.54%) | 423 (2.18%) | 308 (1.48%) | 111 (0.40%) | 163 (2.96%) |  |
| **Injury Category** |  |  |  |  |  | <0.001 |
| transport | 55 (1.30%) | 455 (2.34%) | 1013 (4.88%) | 1294 (4.71%) | 275 (4.99%) |  |
| fall | 1410 (33.40%) | 4411 (22.72%) | 4486 (21.59%) | 5129 (18.68%) | 1090 (19.79%) |  |
| blunt/heavy object | 29 (0.69%) | 259 (1.33%) | 333 (1.60%) | 431 (1.57%) | 79 (1.43%) |  |
| knife/sharp object | 0 (0.00%) | 0 (0.00%) | 0 (0.00%) | 0 (0.00%) | 1 (0.02%) |  |
| knife/sharp object | 54 (1.28%) | 403 (2.08%) | 509 (2.45%) | 796 (2.90%) | 143 (2.60%) |  |
| fire, heat, and hot substances | 251 (5.95%) | 1155 (5.95%) | 495 (2.38%) | 454 (1.65%) | 67 (1.22%) |  |
| asphyxiation | 1 (0.02%) | 1 (0.01%) | 1 (0.00%) | 0 (0.00%) | 1 (0.02%) |  |
| drowning | 2 (0.05%) | 19 (0.10%) | 15 (0.07%) | 17 (0.06%) | 0 (0.00%) |  |
| poisoning | 70 (1.66%) | 407 (2.10%) | 311 (1.50%) | 96 (0.35%) | 156 (2.83%) |  |
| animal contact | 9 (0.21%) | 135 (0.70%) | 216 (1.04%) | 391 (1.42%) | 88 (1.60%) |  |
| unknown/others | 2341 (55.45%) | 12170 (62.68%) | 13395 (64.48%) | 18852 (68.65%) | 3608 (65.50%) |  |
| **Year** |  |  |  |  |  | <0.001 |
| 2018 | 1236 (29.28%) | 5841 (30.08%) | 5509 (26.52%) | 6019 (21.92%) | 920 (16.70%) |  |
| 2019 | 1349 (31.95%) | 6661 (34.31%) | 6289 (30.27%) | 7353 (26.78%) | 1140 (20.70%) |  |
| 2020 | 95 (2.25%) | 458 (2.36%) | 545 (2.62%) | 711 (2.59%) | 203 (3.69%) |  |
| 2021 | 52 (1.23%) | 337 (1.74%) | 407 (1.96%) | 584 (2.13%) | 237 (4.30%) |  |
| 2022 | 490 (11.61%) | 2031 (10.46%) | 2616 (12.59%) | 3576 (13.02%) | 963 (17.48%) |  |
| 2023 | 1000 (23.69%) | 4087 (21.05%) | 5408 (26.03%) | 9217 (33.57%) | 2045 (37.13%) |  |
| **Department** |  |  |  |  |  | <0.001 |
| outpatient/emergency | 4215 (99.83%) | 19141 (98.59%) | 20149 (96.99%) | 26278 (95.70%) | 4958 (90.01%) |  |
| rescue | 4 (0.09%) | 132 (0.68%) | 112 (0.54%) | 66 (0.24%) | 96 (1.74%) |  |
| hospitalized | 3 (0.07%) | 142 (0.73%) | 513 (2.47%) | 1116 (4.06%) | 454 (8.24%) |  |
